# The effect of binaural beats on phonological awareness and cortical connectivity in dyslexic children

**DOI:** 10.1038/s41598-026-56452-2

**Published:** 2026-06-05

**Authors:** Noushin Sangtarash, Mohammad Reza Bigdeli, Jalil Fathabadi, Mohammad Ali Nazari

**Affiliations:** 1 Institute of Cognitive and Brain Sciences, Shahid Beheshti University, Tehran, Iran; 2 Department of Animal Sciences and Marine Biology, Faculty of Life Sciences and Biotechnology, Shahid Beheshti University, Tehran, Iran; 3 Department of Psychology, Shahid Beheshti University, Tehran, Iran; 4 Department of Neuroscience, Faculty of Advanced Technologies in Medicine, Iran University of Medical Sciences, Tehran, Iran

**Keywords:** Binaural beat, Cortical connectivity, Dyslexia, Phonological awareness, Neuroscience, Psychology, Psychology

## Abstract

Developmental dyslexia involves deficits in phonological awareness and cortical connectivity, yet the effects of binaural beats on these domains remain underexplored. This study examined the effects of theta (5 Hz) and beta (15 Hz) binaural beats on phonological processing and EEG coherence in 45 dyslexic children aged 6.5–8.3 years. Participants were assigned to the theta, beta, or control condition and received 12 binaural beat sessions over a period of four weeks. Resting-state EEGs were recorded at baseline, after the binaural beats sessions, and at a 6-week follow-up. Results showed that theta binaural beats significantly enhanced intrahemispheric coherence in frontal and temporal regions, correlating with improved phonological awareness. Beta beats enhanced interhemispheric coherence, particularly between temporal lobes, potentially supporting phonological decoding. The control group showed no significant changes. Correlation analysis revealed that coherence in specific brain regions (e.g., T3–T4, *r* = 0.788, *p* = 0.0013) was significantly associated with performance on phoneme judgment tasks. These findings suggest that frequency-specific binaural beats can enhance neural connectivity and phonological processing. This non-invasive approach shows potential for further exploration of cognitive effects in dyslexic children, though limitations such as small sample size and restricted frequency range necessitate additional research.

## Introduction

Developmental dyslexia is the most prevalent developmental disorder (affects approximately 5–10% of the global population), characterized by an otherwise inexplicable failure to learn to read in children of at least average intelligence. Gender differences have been observed, with a higher incidence reported in males, potentially due to both biological factors and referral biases^1^.

It is most commonly defined as a problem with the decoding of the written word, despite adequate intelligence, education, and socioeconomic status, with such processing difficulties leading to the formulation of the phonological core deficit hypothesis of dyslexia^2^.

Recent research has shown that children with dyslexia have impairments in both phonological skills and working memory. However, developmental dyslexia is increasingly seen as a complex neurodevelopmental disorder involving abnormal coordination of multiple brain networks, rather than just a phonological problem. Functional connectivity studies using resting-state and task-based imaging have consistently shown decreased coordination between frontal and posterior language areas, especially between the left inferior frontal gyrus (IFG) and the left fusiform gyrus (FFG), indicating that differences in neural network integration may cause reading difficulties in dyslexia across both task and resting conditions^3^. Additionally, broader disruptions in connectivity have been observed between language networks and executive control circuits, suggesting that cognitive processes beyond phonology, such as attention and cross-modal integration, are involved. These neurobiological findings support systematic reviews that show altered coordination across multiple brain networks, including language, executive function, and default mode systems, contributes to the variability in reading performance among individuals with dyslexia^4^.

Recent conceptualizations increasingly frame dyslexia not as a singular deficit, but as a complex, multifactorial condition arising from the intricate interplay of diverse cognitive and neurobiological components. This integrative perspective posits that difficulties in reading acquisition are influenced by a confluence of factors, including, but not limited to, phonological processing, perceptual (e.g., visual and auditory) processing, attentional control, temporal processing speed, and underlying neurobiological differences in brain structure and function^5,6^.

A growing body of literature supports this holistic view. For instance, studies have demonstrated the significant role of phonological deficits in dyslexia, while others highlight the contribution of visual processing challenges, such as difficulties with rapid naming or visual attention^3^. Furthermore, research has implicated temporal processing deficits, including those related to auditory beat perception, in the symptomatology of dyslexia. The neurobiological underpinnings are also extensively studied, revealing patterns of altered connectivity and activation in brain regions crucial for reading, such as the temporo-parietal and occipito-temporal pathways^1^.

This multifactorial perspective suggests that dyslexic reading difficulties are better understood as the result of widespread neural network disturbances affecting phonological, attentional, and cross‑hemispheric integration processes, reinforcing the rationale to explore intervention strategies that target broad network modulation rather than narrow symptom domains alone.

Phonological awareness, defined as a metalinguistic skill to reflect upon and manipulate the sound structure of spoken words, plays a significant role in reading development in the lower grades of primary education^7^.

Individual differences in children’s “phonological awareness” (their awareness of the sound structure of words as measured by behavioral tasks) are related to their progress in reading and spelling development, and this relationship has been found in all languages so far studied. It is important to note that many factors other than sensory/neural processing also determine the child’s development of phonological awareness^8^.

While many factors will play a role in developing a high‐quality phonological system, the efficiency of children’s sensory/neural processing of acoustic information appears to be a key source of individual differences in phonological learning. The nature of the sensory/neural processing that may govern individual differences in the quality of children’s phonological representations is the focus of this paper^9^.

Functional connectivity is a way of measuring the strength of the connection between different structures. Most studies exploring brain activity in children with learning disorders using EEGs have been based on EEG power analysis, and few have focused on functional connectivity measures.^10^.

Brain coherence is a critical component in efficient information processing, allowing various regions of the brain to communicate effectively during complex tasks like reading^11^.

Studies show that dyslexia is associated with differences in neural oscillation patterns and synchronization, which can be measured as brain coherence^12^. Studies reported that dyslexic children show decreased coherence in the theta and beta frequency bands, which are critical for phonological processing and attention^13^. These findings are supported by Arns et al. (2007), who found that dyslexic children often exhibit reduced beta coherence, associated with difficulties in focusing and processing linguistic information^14^.

Research indicates that dyslexic children generally exhibit reduced interhemispheric coherence in the beta band, particularly between regions critical for language processing, such as the left and right temporal areas. This reduced coherence may contribute to difficulties in cross-hemispheric communication, which is essential for integrating phonological and visual information during reading tasks^12,15,16^.

In contrast, intrahemispheric coherence in dyslexic children tends to be increased within the left hemisphere in both beta and theta bands, particularly when engaged in language and phonological tasks. This elevated left-hemisphere coherence may serve as a compensatory mechanism to support language processing in the face of interhemispheric deficits. The increase in left-hemispheric theta coherence is especially associated with attempts to recruit memory and language processing areas. However, it may not fully compensate for the interhemispheric deficits that impact efficient reading^16^.

These coherence patterns reduced interhemispheric beta coherence, combined with increased intrahemispheric theta and beta coherence in the left hemisphere, highlighting the neurophysiological challenges dyslexic children face. They suggest that interventions focusing on improving cross-hemispheric connectivity could potentially support more integrated and efficient reading pathways^17^.

Binaural beats are an auditory phenomenon created when two slightly different frequencies are presented to each ear, and the brain perceives a third, distinct frequency, which is the mathematical difference between the two^18^. For example, if one ear hears a 300 Hz tone and the other hears a 310 Hz tone, the brain detects a 10 Hz beat. They are also purported to influence brainwave activity, which can lead to relaxation, focus, and improved mood. Binaural beats are purported to cause relaxation, increase cognitive function, and improve the quality of sleep^19,20^. It is claimed that these effects are achieved because the brain tends to entrain its electric activity with the frequency of the perceived beat via a process known as the frequency-following response^18^.

There has been research on binaural beats to make use of them in various conditions, such as anxiety, pain, and improved cognition^21^. For example, studies have established that binaural beats alleviate preoperative anxiety and improve attention in ADHD patients^22,23^. The findings are inconclusive, and larger and stricter trials must be done to establish and make standardized recommendations for them^24^.

Auditory training-based treatments try to improve the ability of phonological processing, which is a foundational aspect of reading. Computer programs like Fast ForWord and others target skills in auditory discrimination, memory, and sequencing. Despite some studies reporting increased phonological awareness and reading skill after such treatments, outcomes are variable, and the efficacy of such treatments is debatable. Above all, binaural beats as an application to enhance phonological awareness in children with dyslexia is quite an untapped area of research, and thus, the potential significance and novelty of our study are highlighted.

Binaural beats are classified by the frequency of the beat, which can range from delta (1–4 Hz) to gamma (30–100 Hz). The most commonly studied binaural beats in the context of cognitive enhancement are those in the alpha (8–12 Hz), theta (4–8 Hz), and beta (12–30 Hz) ranges. These frequencies are associated with different mental states and cognitive processes^19,24^.

Recent evidence has increasingly investigated the potential of binaural beats (BBs) to affect a range of cognitive functions, including attention, memory, and mood regulation. Within the field of attention, the gamma range BBs (35–40 Hz) have been found to optimize sustained attention and cognitive engagement, as evidenced through better behavioral performance and EEG markers of entrainment^25^.

Memory processes have also been targeted in BB research. One study noted that theta frequency BBs (5–8 Hz) were able to improve cognition scores in patients with Alzheimer’s disease. EEG measures showed that there was increased theta power at the frontal and central electrodes when participants were involved in language and orientation tasks^26^. However, in healthy populations, combinations of BB (particularly calming background music) suggested increased left frontal beta activity and relative theta power, but were also associated with increased attentional regulation and memory encoding^27^. These electrophysiological shifts in the EEG data would suggest that BBs could lead to more ideal neural conditions for memory formation and consolidation.

For emotional regulation, while low frequency BBs (≤ 10 Hz) show the potential for lowered anxiety and increased mood states (though results are mixed and possibly impacted by placebo factors)^28^. There is more solid evidence for gamma frequency BBs, especially at 40 Hz, which have been shown to positively affect alertness and positive affect. EEG connectivity studies suggest that the brain areas involved in connectivity show modulation for frontal-central and parietal areas, some perhaps linked to arousal aspects of emotional processing^29^.

While binaural beats have been researched for their effects on various cognitive functions, including attention, memory, and mood regulation^30^, studies specifically focusing on dyslexia remain limited.

The difficulties with phonological processing in dyslexia have been linked to abnormalities in auditory processing systems and neural activation patterns in areas of the brain responsible for reading and language processing, such as the left temporal cortex, left parietal cortex, and inferior frontal gyrus. These individuals often show asynchrony in neural firing between the two hemispheres of the brain, particularly in auditory processing regions^31^. The idea behind using binaural beats as an intervention is that they might promote neural entrainment, a process in which brainwaves synchronize to the frequency of the binaural beat, potentially improving the neural systems responsible for phonological processing^18,32^. Studies on binaural beats in general populations have shown that they can synchronize brainwave activity across different cortical regions, especially in areas involved in auditory processing. By promoting neural synchronization, binaural beats could help improve the brain’s ability to differentiate and process phonemes, a crucial skill for reading^31^.

Previous studies have not examined the effect of binaural beats on phonological deficit improvement or the relationship of phonological improvement to improvements in brain connectivity after binaural beats, one of the core factors underlying phonological processing deficits in children with dyslexia. Additionally, the duration and frequency of the sessions of the binaural beat have been cited as limitations of previous studies. To address this, binaural beats were provided for a greater length of time in our study, consisting of 12 sessions of 10 min each. This was anticipated to enhance the effectiveness of the binaural beat by optimizing the duration of exposure, which is extremely critical to maximizing its effect on phonological and cortical connectivity improvement.

On the other hand, despite promising theoretical and experimental findings, the research on binaural beats and dyslexia is still in its early stages.

The present study aims to investigate whether binaural beats in the theta (5 Hz) and beta (15 Hz) frequency bands can enhance cortical coherence and, consequently, improve phonological processing skills (phonological awareness) in children with dyslexia.

The novelty of this study lies in the targeted investigation of frequency-specific binaural beats for dyslexia. This study is novel compared to previous research in that it employs a controlled experimental methodology to identify the effects of binaural beats on phonological processing and cortical connectivity. Using EEG-based coherence analysis, this study will provide novel insights into the neurophysiological mechanisms underlying dyslexia.

## Results

Statistical analysis performed throughout this study focused on the influence of theta and beta binaural beats on intrahemispheric and interhemispheric coherence, as well as their correlation with phonological awareness abilities. Assumption checks indicated that EEG coherence measures met normality and homogeneity of variance assumptions, whereas phonological awareness scores did not meet normality assumptions. No significant baseline differences were observed between groups for phonological awareness scores or EEG coherence measures at pre-test, as assessed using the Kruskal–Wallis test (all *p*-values > 0.05).

### Effects of binaural beats on phonological awareness

As phonological awareness scores did not meet the assumption of normality, non-parametric statistical analyses were employed. Within-group changes in phonological awareness across the three time points (pre-test, post-test, and follow-up) were examined using the Friedman test. A significant effect of time was observed in the beta binaural beat group, χ^2^(2) = 9.14, *p* = 0.010, indicating a significant change in phonological awareness scores across the three time points. Similarly, the theta binaural beat group showed a significant time effect, χ^2^(2) = 7.86, *p* = 0.020. In contrast, no significant change across time was observed in the control group, χ^2^(2) = 1.09, *p* = 0.580.

To compare the magnitude of phonological awareness improvement between groups, change scores (post-test vs. pre-test) were calculated and analyzed using the Kruskal–Wallis test. The analysis showed a significant group effect, H(2) = 8.32, *p* = 0.016. Post hoc Dunn–Bonferroni comparisons indicated that both the beta binaural beat group (*p* = 0.012) and the theta binaural beat group (*p* = 0.028) showed significantly greater improvements in phonological awareness compared to the control group. No significant difference was found between the beta and theta binaural beat groups (*p* = 0.410). No significant decline in phonological awareness was observed at follow-up in either stimulation group, suggesting that the improvements were maintained over time (Fig. 13).

### Effects of binaural beats on cortical connectivity

#### Within-group changes in cortical connectivity

Within-group changes in cortical connectivity were examined across pre-test, post-test, and follow-up sessions. Analyses focused on left intrahemispheric, right intrahemispheric, and interhemispheric coherence within the stimulation-specific frequency bands.

In the theta binaural beat group, Follow-up analyses of the significant Time × Group interaction showed significant increases in coherence from pre-test to post-test, predominantly within left intrahemispheric fronto-parietal and fronto-temporal networks, including electrode pairs such as F3–P3 and F3–T3 (*p* < 0.05, FDR-corrected). These changes remained stable at follow-up.

In the beta binaural beat group, the most pronounced effects were observed in interhemispheric coherence, particularly in temporal regions (e.g., T3–T4), as well as in bilateral fronto-temporal connections (*p* < 0.05, FDR-corrected).

In contrast, the control group did not exhibit significant within-group changes in cortical connectivity in either the beta or theta frequency bands across any electrode pairs (all *p*-values > 0.05).

#### Between-group comparisons of connectivity change

A mixed-design repeated-measures ANOVA with Time (pre-test, post-test, follow-up) as the within-subject factor and Group (beta, theta, control) as the between-subject factor showed a significant Time × Group interaction for interhemispheric coherence, *F*(4, 84) = 4.12, *p* = 0.004, η2p = 0.17, indicating differential patterns of change in interhemispheric connectivity across groups over time.

To further characterize the direction and magnitude of group differences underlying this interaction in interhemispheric coherence, coherence change scores (Δ = post-test − pre-test) were calculated. As assumptions of normality were not consistently met, between-group comparisons of these change scores were conducted using the Kruskal–Wallis test. A significant group effect was observed for interhemispheric coherence change, *H*(2) = 8.21, *p* = 0.016. Post hoc Dunn–Bonferroni comparisons indicated that the beta binaural beat group exhibited significantly larger increases in interhemispheric coherence compared to both the theta binaural beat group and the control group (adjusted *p* < 0.05).

In addition to interhemispheric effects, between-group differences in left intrahemispheric coherence change were examined using change scores (Δ = post-test − pre-test). A significant group effect was observed, *H*(2) = 6.94, *p* = 0.031. Post hoc Dunn–Bonferroni comparisons showed that increases in left intrahemispheric coherence were significantly greater in the theta binaural beat group than in the beta binaural beat group (adjusted *p* = 0.032) and the control group (adjusted *p* = 0.018).

Consistent with the significant Time × Group interaction observed in the repeated-measures ANOVA, these findings demonstrate frequency-specific modulation of cortical connectivity, with theta-frequency stimulation preferentially enhancing localized left-hemispheric networks and beta-frequency stimulation primarily influencing cross-hemispheric integration.

These findings demonstrate frequency-specific modulation of cortical connectivity, with theta stimulation preferentially enhancing localized left-hemispheric networks and beta stimulation primarily influencing cross-hemispheric integration.

### Associations between cortical connectivity and phonological awareness

To investigate the relationship between neural connectivity and behavioral performance, Pearson correlation analyses were conducted to assess the relationship between changes in cortical coherence and phonological awareness scores within each group. False Discovery Rate (FDR) correction was applied to account for multiple correlations.

In the beta binaural beat group, phonological awareness improvements were correlated with increases in left intrahemispheric beta-band coherence (r = 0.83, *p* = 0.001, n = 15) and interhemispheric beta-band coherence (r = 0.74, p = 0.003, n = 15). A positive correlation was also observed with right intrahemispheric coherence (r = 0.72, *p* = 0.005, n = 15).

In the theta binaural beat group, phonological awareness scores were correlated with interhemispheric theta-band coherence (r = 0.65, *p* = 0.010, n = 15). Correlations with left and right intrahemispheric theta coherence were weaker and did not consistently reach statistical significance after correction for multiple comparisons.

In the control group, no significant correlations were observed between phonological awareness scores and cortical coherence measures in either frequency band (all *p*-values > 0.05).

In summary, these results indicate that improvements in phonological awareness are associated with frequency-specific patterns of cortical connectivity following binaural beat stimulation, without implying a direct causal relationship (Figs. 1, 2, 3, 4, 5, 6, 7, 8, 9, 10, 11, 12).

**Fig. 1:**
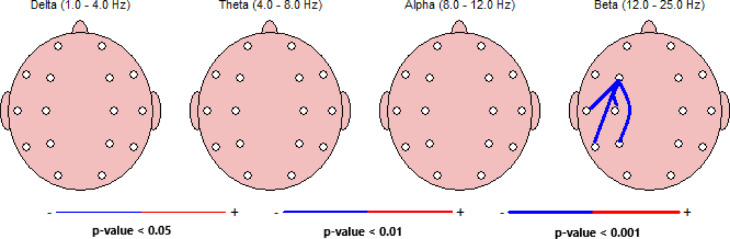
Left hemispheric EEG coherency (*p*-values) for the beta-band in the beta binaural beat group: FFT left hemispheric coherence (p-values) of the left hemisphere for beta band frequency across paired EEG electrodes(FP1-F3, FP1-C3, FP1-P3, FP1-O1, FP1-F7, FP1-T3, FP1-T5, F3-C3, F3-P3, F3-O1, F3-F7, F3-T3, F3-T5, C3-P3, C3-O1, C3-F7, C3-T3, C3-T5, P3-O1, P3-F7, P3-T3, P3-T5, O1-T3, O1-T5, F7-T3, F7-T5, T3-T5) in the beta binaural beat group. Line thickness indicates the level of statistical significance (p-value), with thicker lines representing more significant changes. Decreases in coherence are shown in blue, whereas increases in coherence are shown in red. Significant changes were observed in coherence values, particularly at F3-C3, F3-P3, F3-T3, and F3–T5 electrode pairs, indicating potential effects of beta binaural beat on left intrahemispheric coherence in the beta frequency band.

**Fig. 2:**
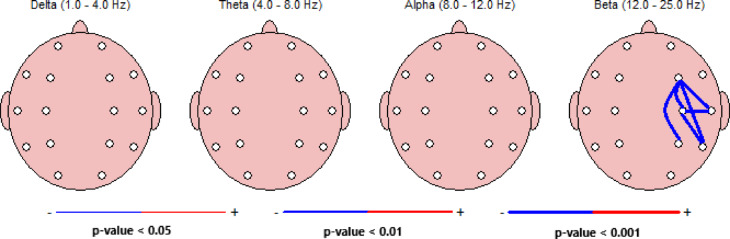
Right hemispheric EEG coherency (p-values) for the beta-band in the beta binaural beat group: FFT right hemispheric coherence (p-values) of the right hemisphere for beta band frequency across paired EEG electrodes (FP2-F4, FP2-C4, FP2-P4, FP2-O2, FP2-F8, FP2-T4, FP2-T6, F4-C4, F4-P4, F4-O2, F4-F8, F4-T4, F4-T6, C4-P4, C4-O2, C4-F8, C4-T4, C4-T6, P4-O2, P4-F8, P4-T4, P4-T6, O2-T4, O2-T6, F8-T4, F8-T6, T4-T6) in the beta binaural beat group. Line thickness indicates the level of statistical significance (p-value), with thicker lines representing more significant changes. Decreases in coherence are shown in blue, whereas increases in coherence are shown in red. Significant changes were observed in coherence values, particularly at F4-P4, F4-T4, F4-T6, C4-T4, and C4-T6 electrode pairs, indicating potential effects of beta binaural beat on right intrahemispheric coherence in the beta frequency band.

**Fig. 3:**
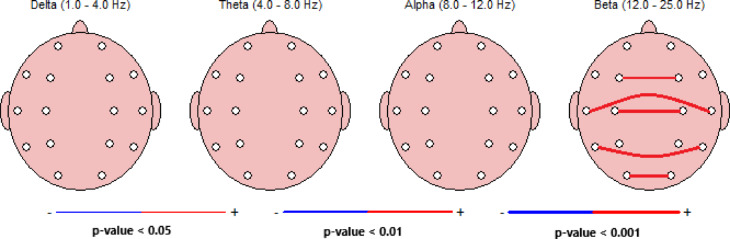
Interhemispheric EEG coherency (p-values) for the beta-band in the beta binaural beat group: FFT interhemispheric coherence (p-values) of beta band frequency across paired EEG electrodes (FP1–FP2, C3–C4, O1–O2, T3–T4, F3–F4, P3–P4, F7–F8, T5–T6) in the beta binaural beat group. Line thickness indicates the level of statistical significance (p-value), with thicker lines representing more significant changes. Decreases in coherence are shown in blue, whereas increases in coherence are shown in red. Significant changes were observed in coherence values, particularly at C3–C4, O1-O2, T3-T4, F3-F4, and T5–T6 electrode pairs, indicating potential effects of beta binaural beat on interhemispheric coherence in the beta frequency band.

**Fig. 4:**
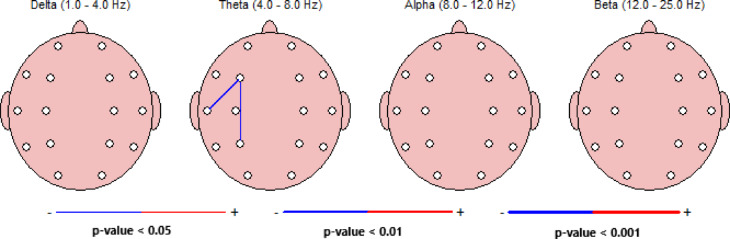
Left hemispheric EEG coherency (p-values) for theta-band in the theta binaural beat group: FFT left hemispheric coherence (p-values) of the left hemisphere for theta band frequency across paired EEG electrodes(FP1-F3, FP1-C3, FP1-P3, FP1-O1, FP1-F7, FP1-T3, FP1-T5, F3-C3, F3-P3, F3-O1, F3-F7, F3-T3, F3-T5, C3-P3, C3-O1, C3-F7, C3-T3, C3-T5, P3-O1, P3-F7, P3-T3, P3-T5, O1-T3, O1-T5, F7-T3, F7-T5, T3-T5) in the theta binaural beat group. Line thickness indicates the level of statistical significance (p-value), with thicker lines representing more significant changes. Decreases in coherence are shown in blue, whereas increases in coherence are shown in red. Significant changes were observed in coherence values, particularly at F3-P3 and F3–T3 electrode pairs, indicating potential effects of theta binaural beat on left intrahemispheric coherence in the theta frequency band.

**Fig. 5:**
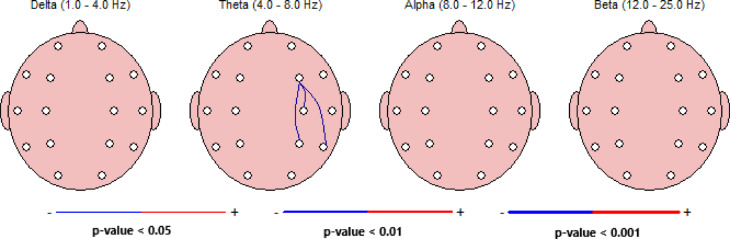
Right hemispheric EEG coherency (p-values) theta-band in the theta binaural beat group: FFT right hemispheric coherence (p-values) of the right hemisphere for theta band frequency across paired EEG electrodes(FP2-F4, FP2-C4, FP2-P4, FP2-O2, FP2-F8, FP2-T4, FP2-T6, F4-C4, F4-P4, F4-O2, F4-F8, F4-T4, F4-T6, C4-P4, C4-O2, C4-F8, C4-T4, C4-T6, P4-O2, P4-F8, P4-T4, P4-T6, O2-T4, O2-T6, F8-T4, F8-T6, T4-T6) in the theta binaural beat group. Line thickness indicates the level of statistical significance (p-value), with thicker lines representing more significant changes. Decreases in coherence are shown in blue, whereas increases in coherence are shown in red. Significant changes were observed in coherence values, particularly at F4-C4, F4-P4, and F4-T6 electrode pairs, indicating potential effects of theta binaural beat on right intrahemispheric coherence in the theta frequency band.

**Fig. 6:**
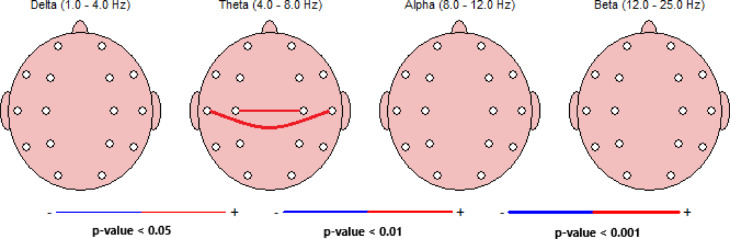
Interhemispheric EEG coherency (p-values) for theta-band in the theta binaural beat group: FFT interhemispheric coherence (p-values) of theta band frequency across paired EEG electrodes (FP1–FP2, C3–C4, O1–O2, T3–T4, F3–F4, P3–P4, F7–F8, T5–T6) in the theta binaural beat group. Line thickness indicates the level of statistical significance (p-value), with thicker lines representing more significant changes. Decreases in coherence are shown in blue, whereas increases in coherence are shown in red. Significant changes were observed in coherence values, particularly at C3–C4 and T3–T4 electrode pairs, indicating potential effects of theta binaural beat on interhemispheric coherence in the theta frequency band.

**Fig. 7:**
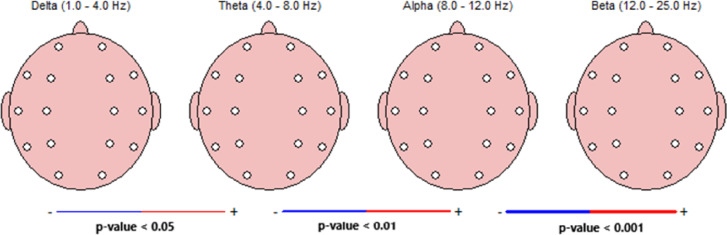
Left hemispheric EEG coherency (p-values) for the beta-band in the control group: FFT left hemispheric coherence (p-values) of the left hemisphere for beta band frequency across paired EEG electrodes (FP1-F3, FP1-C3, FP1-P3, FP1-O1, FP1-F7, FP1-T3, FP1-T5, F3-C3, F3-P3, F3-O1, F3-F7, F3-T3, F3-T5, C3-P3, C3-O1, C3-F7, C3-T3, C3-T5, P3-O1, P3-F7, P3-T3, P3-T5, O1-T3, O1-T5, F7-T3, F7-T5, T3-T5) in the control group. Line thickness indicates the level of statistical significance (p-value), with thicker lines representing more significant changes. Decreases in coherence are shown in blue, whereas increases in coherence are shown in red. No significant changes were observed in coherence values, indicating that the effects observed in the experimental groups could be attributed to the binaural beat and not to exposure as a general phenomenon.

**Fig. 8:**
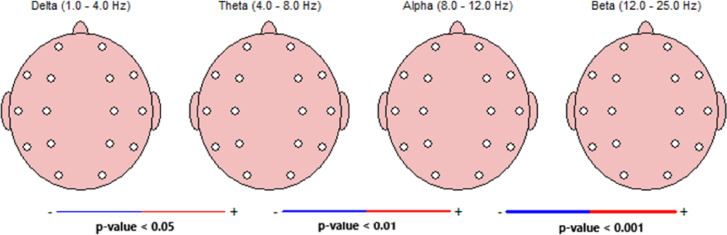
Right hemispheric EEG coherency (p-values) for the beta-band in the control group: FFT right hemispheric coherence (p-values) of the right hemisphere for beta band frequency across paired EEG electrodes (FP2-F4, FP2-C4, FP2-P4, FP2-O2, FP2-F8, FP2-T4, FP2-T6, F4-C4, F4-P4, F4-O2, F4-F8, F4-T4, F4-T6, C4-P4, C4-O2, C4-F8, C4-T4, C4-T6, P4-O2, P4-F8, P4-T4, P4-T6, O2-T4, O2-T6, F8-T4, F8-T6, T4-T6)in the control group. Line thickness indicates the level of statistical significance (p-value), with thicker lines representing more significant changes. Decreases in coherence are shown in blue, whereas increases in coherence are shown in red. No significant changes were observed in coherence values, indicating that the effects observed in the experimental groups could be attributed to binaural beat, rather than to exposure as a general phenomenon.

**Fig. 9:**
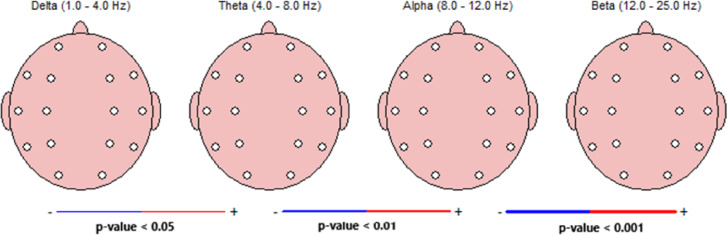
Interhemispheric EEG coherency (p-values) for the beta-band in the control group: FFT interhemispheric coherence (p-values) of beta band frequency across paired EEG electrodes (FP1–FP2, C3–C4, O1–O2, T3–T4, F3–F4, P3–P4, F7–F8, T5–T6) in the control group. Line thickness indicates the level of statistical significance (p-value), with thicker lines representing more significant changes. Decreases in coherence are shown in blue, whereas increases in coherence are shown in red. No significant changes were observed in coherence values, indicating that the effects observed in the experimental groups could be attributed to binaural beat, rather than to exposure as a general phenomenon.

**Fig. 10:**
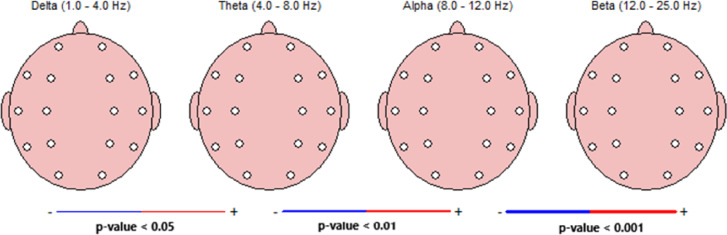
Left hemispheric EEG coherency (p-values) for theta-band in the control group: FFT left hemispheric coherence (p-values) of the left hemisphere for theta band frequency across paired EEG electrodes(FP1-F3, FP1-C3, FP1-P3, FP1-O1, FP1-F7, FP1-T3, FP1-T5, F3-C3, F3-P3, F3-O1, F3-F7, F3-T3, F3-T5, C3-P3, C3-O1, C3-F7, C3-T3, C3-T5, P3-O1, P3-F7, P3-T3, P3-T5, O1-T3, O1-T5, F7-T3, F7-T5, T3-T5) in the control group. Line thickness indicates the level of statistical significance (p-value), with thicker lines representing more significant changes. Decreases in coherence are shown in blue, whereas increases in coherence are shown in red. No significant changes were observed in coherence values, indicating that the effects observed in the experimental groups could be attributed to binaural beat, rather than to exposure as a general phenomenon.

**Fig. 11:**
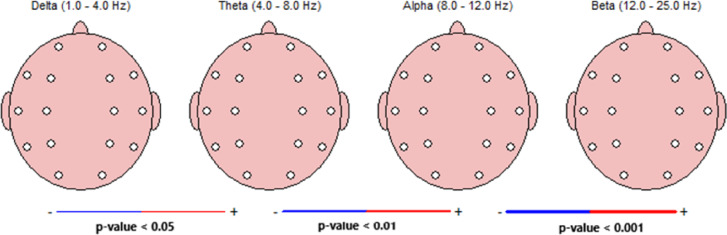
Right hemispheric EEG coherency (p-values) for the theta-band in the control group: FFT right hemispheric coherence (p-values) of the right hemisphere for theta band frequency across paired EEG electrodes(FP2-F4, FP2-C4, FP2-P4, FP2-O2, FP2-F8, FP2-T4, FP2-T6, F4-C4, F4-P4, F4-O2, F4-F8, F4-T4, F4-T6, C4-P4, C4-O2, C4-F8, C4-T4, C4-T6, P4-O2, P4-F8, P4-T4, P4-T6, O2-T4, O2-T6, F8-T4, F8-T6, T4-T6) in the control group. Line thickness indicates the level of statistical significance (p-value), with thicker lines representing more significant changes. Decreases in coherence are shown in blue, whereas increases in coherence are shown in red. No significant changes were observed in coherence values, indicating that the effects observed in the experimental groups could be attributed to binaural beat, rather than to exposure as a general phenomenon.

**Fig. 12:**
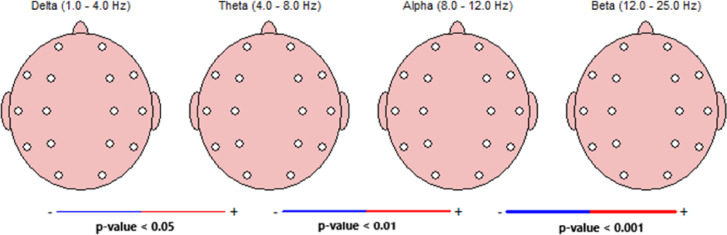
Interhemispheric EEG coherency (p-values) for the theta-band in the control group: FFT interhemispheric coherence (p-values) of theta band frequency across paired EEG electrodes (FP1–FP2, C3–C4, O1–O2, T3–T4, F3–F4, P3–P4, F7–F8, T5–T6) in the control group. Line thickness indicates the level of statistical significance (p-value), with thicker lines representing more significant changes. Decreases in coherence are shown in blue, whereas increases in coherence are shown in red. No significant changes were observed in coherence values, indicating that the effects observed in the experimental groups could be attributed to the binaural beat and not to exposure as a general phenomenon.

**Fig. 13:**
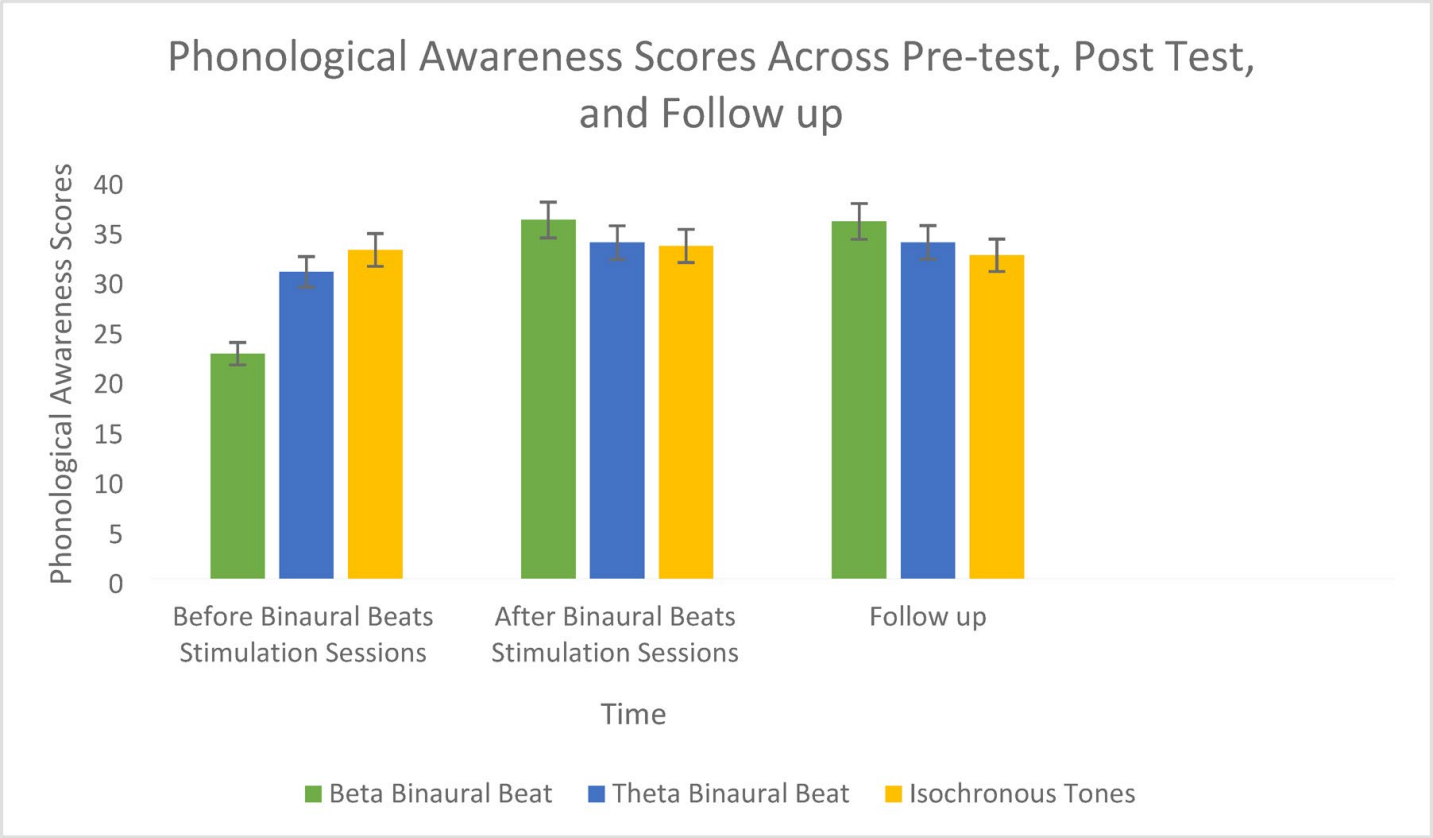
Mean phonological awareness scores across assessment time points for the three groups. The figure indicates changes in phonological awareness scores measured before binaural beat stimulation (pre-test), after completion of the stimulation sessions (post-test), and at follow-up for the beta binaural beat, theta binaural beat, and isochronous tone (control) groups. Bars represent group mean scores, and error bars indicate the standard error of the mean (SEM). Both binaural beat groups show a clear increase in phonological awareness from pre-test to post-test, with gains maintained at follow-up, whereas the control group does not exhibit statistically meaningful changes across time points. These patterns are consistent with the statistical analyses, indicating significant time-related improvements in the stimulation groups but not in the control condition.

**Fig. 14:**
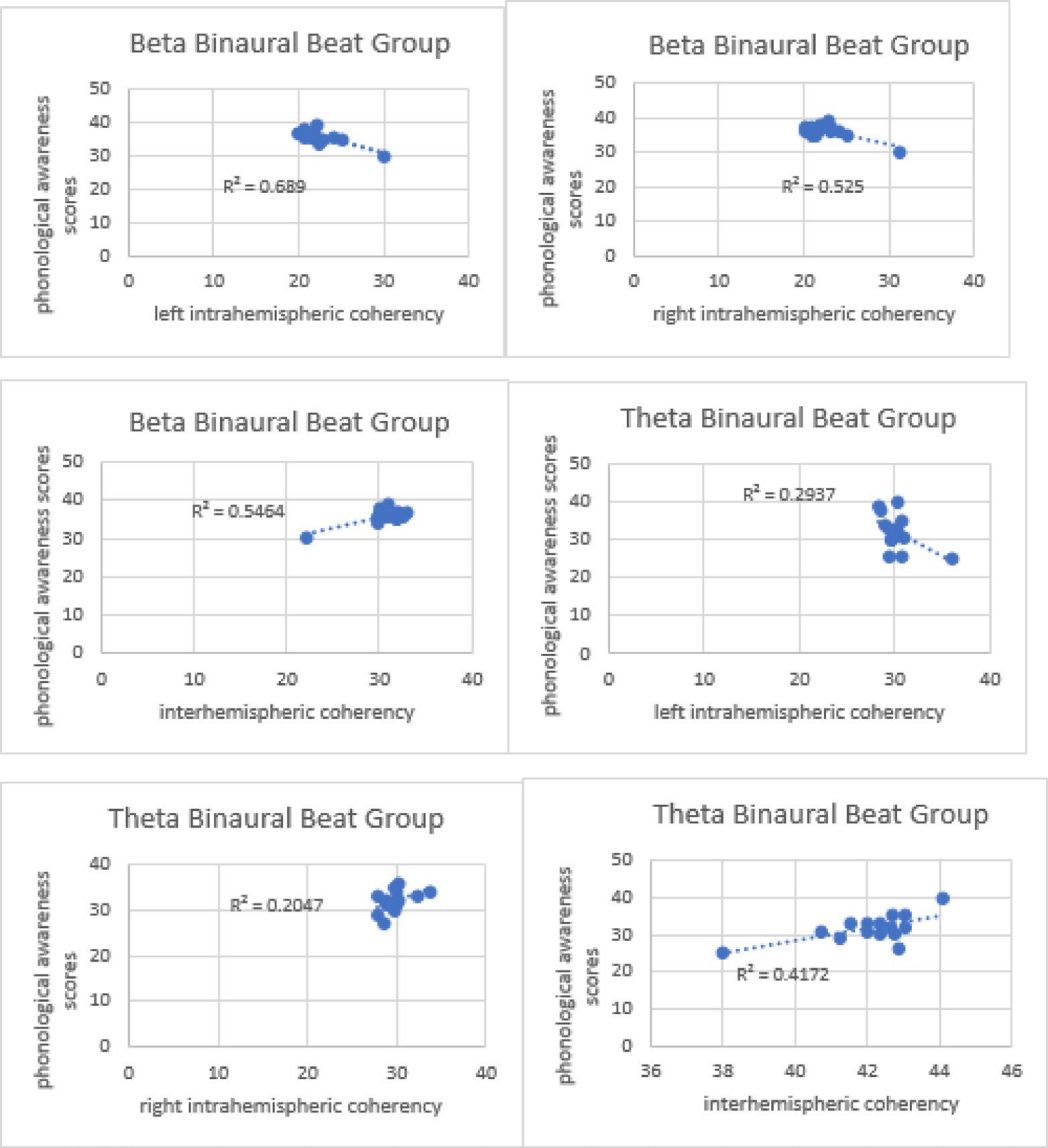

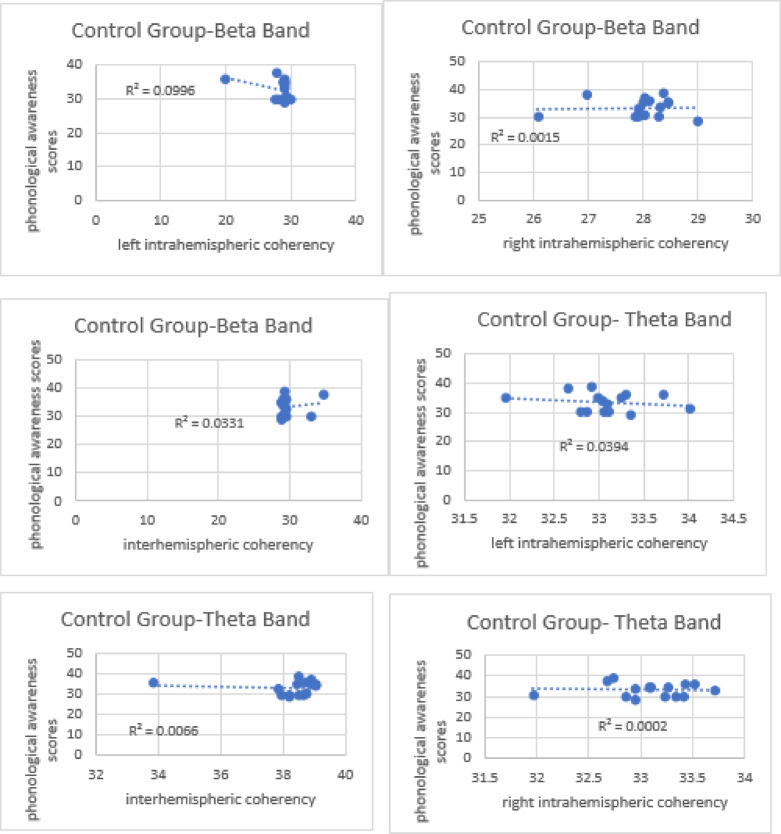
Correlations between phonological awareness and EEG coherency in beta, theta, and control binaural beats groups: Scatter plots indicating the correlation between phonological awareness scores and brain coherency measures (left intrahemispheric coherency, right intrahemispheric coherency, and interhemispheric coherency) in three groups of children with dyslexia (n = 15 per group): a beta binaural beats experimental group, a theta binaural beats experimental group, and a control group. Coherence was assessed in both beta and theta frequency bands. In the beta binaural beats group, both left intrahemispheric coherency and interhemispheric coherency for beta band frequency showed strong positive correlations with phonological awareness scores (R^2^ = 0.689 and R^2^ = 0.5464). In contrast, right intrahemispheric coherency for the beta binaural beat group indicated a moderate correlation (R^2^ = 0.525). In the theta binaural beats group, interhemispheric coherency for theta band frequency demonstrated a moderate positive correlation (R^2^ = 0.4172), with weaker correlation for left and right intrahemispheric for theta band frequency (R^2^ = 0.2937and R^2^ = 0.2047). In the control group, no significant correlations were observed between brain coherency measures and phonological awareness scores in either frequency band (R^2^ < 0.04for all comparisons).

These findings demonstrate that theta and beta binaural beats can enhance both EEG coherence and phonological awareness substantially in children with dyslexia (Figs. 13, 14).

## Discussion

This study was as an exploratory investigation to examine the potential of theta and beta binaural beat stimulation in improving phonological awareness and cortical connectivity in children with dyslexia. While our findings indicate promising improvements in phonological awareness and specific patterns of neural reorganization, it is crucial to interpret these results within an exploratory framework. The observed effects, particularly concerning neural synchronization and their correlation with phonological performance, should be considered hypothesis-generating rather than definitive evidence of a clinically validated treatment. Theses preliminary findings suggest potential mechanisms through which binaural beat stimulation might influence cognitive processing relevant to dyslexia, warranting further investigation.

Consistent with the significant Time × Group interaction observed in the repeated-measures ANOVA, the pattern of interhemispheric coherence changes differed across groups, with beta binaural beats showing the most pronounced and sustained increases over time, theta binaural beats showing more selective effects, and no systematic changes observed in the control group.

Theta binaural beats significantly increased intrahemispheric coherence, particularly in fronto-parietal and fronto-temporal regions such as F3–P3 (*p* = 0.047), F3–T5 (*p* = 0.027), and F4–T6 (*p* = 0.023). These results are consistent with prior research linking theta oscillations to working memory and phonological processing, which are typically impaired in dyslexic children. The observed increase in parietal and temporal connectivity suggests enhanced sensorimotor integration and auditory processing, which are essential for phonological awareness. However, some regions, including C3–P3 (*p* = 0.989) and C4–T6 (*p* = 0.881), did not show significant change, indicating that the theta binaural beat’s effects may be localized rather than widespread. In addition, theta binaural beat was associated with selective increases in interhemispheric coherence, primarily in temporal regions (T3–T4, *p* = 0.000), which are essential for transferring phonological information between hemispheres. As the corpus callosum mediates such integration, these results suggest that theta beats enhance the coordination of language and auditory processes. Increased coherence in C3–C4 (*p* = 0.008) further points to a potential benefit for phonological decoding and reading fluency. By contrast, occipital (O1–O2, *p* = 0.303) and parietal (P3–P4, *p* = 0.295) regions did not show significant change, suggesting that theta effects are more salient for auditory–language pathways than for visual–spatial processing.

Beta binaural beats also produced noticeable increases in intrahemispheric coherence, particularly in fronto-central and fronto-temporal areas, including F3–C3 (*p* = 0.001), F3–P3 (*p* = 0.000), and F3–T3 (*p* = 0.000). These regions play an important role in phonological processing and motor control, both of which are often compromised in dyslexia. Enhanced coherence in these networks suggests that beta frequencies could support language generation and reading-related cognitive processes. Further increases in F4–C4 (*p* = 0.000), F4–T4 (*p* = 0.000), and F4–T6 (*p* = 0.000) reinforce the role of beta oscillations in facilitating auditory–motor integration. Yet, some posterior areas, such as C4–P4 (*p* = 0.103) and P4–T6 (*p* = 0.583), did not show significant increases, indicating that beta effects are more pronounced in anterior cortical regions. Beta binaural beat also enhanced interhemispheric coherence, notably in T3–T4 (*p* = 0.000), which is consistent with its role in phonological awareness and auditory processing. Additional increases in F3–F4 (*p* = 0.002) and C3–C4 (*p* = 0.001) highlight strengthened connections in phonological and executive control regions. Unlike the theta binaural beat, the beta binaural beat also yielded moderate but significant increases in the occipital (O1–O2, *p* = 0.016) and parietal (P3–P4, p = 0.002) regions, indicating a more distributed cortical effect.

Importantly, the control group that did not receive binaural beat showed no significant intrahemispheric or interhemispheric changes (all *p*-values > 0.05), supporting the interpretation that the observed effects were associated with frequency-specific stimulation rather than passive exposure. Post-hoc analyses further supported these results. Additional analyses suggested that theta beats primarily enhanced intrahemispheric coherence in fronto-parietal and fronto-temporal areas, whereas beta beats exerted stronger effects on interhemispheric connectivity, particularly in temporal lobes. FDR corrections indicated that the significant effects remained after adjustment for multiple comparisons, thereby reducing the risk of Type I error.

Correlational analyses showed that binaural beats were functionally relevant to phonological awareness skills. In particular, beta-frequency correlated with phonological awareness scores, with left intrahemispheric and interhemispheric coherence showing the positive relationships. Right intrahemispheric coherence for beta band frequency contributed as well, but to a lesser degree. The theta binaural beat also showed correlations between left intrahemispheric and interhemispheric coherence and phonological awareness, while right intrahemispheric theta coherence had weaker associations. These findings suggest that while both frequencies support phonological processing and lead to comparable behavioral improvements in phonological awareness, the beta binaural beat was associated with more widespread neural connectivity changes, particularly at the interhemispheric level. By contrast, the control group displayed no meaningful correlations, reinforcing the specificity of the observed effects. Notably, follow-up data collected six weeks post-experiment revealed that no significant decline was observed at follow-up, suggesting maintenance of effects, although the study may have been underpowered to detect subtle changes. suggesting that binaural beats can induce durable neural adaptations relevant for phonological training.

In summary, the present study demonstrates that theta- and beta-frequency binaural beats enhance intra- and interhemispheric connectivity in dyslexic children, particularly within frontal and temporal regions related to phonological processing and working memory. Theta binaural beat had a greater effect on temporal networks critical for auditory–language integration, whereas Beta binaural beats may more engage neural networks related to phonological decoding and executive control, even though behavioral gains in phonological awareness did not differ significantly from those observed in the theta condition. The absence of changes in the control group further confirms that effects were driven by frequency-specific auditory stimulation. The findings suggest that binaural beats may be associated with changes in neural synchrony and phonological processing. While the present study demonstrates the effects of frequency-specific binaural beat on neural coherence and phonological awareness in children with dyslexia, these findings should be interpreted within the wider scientific context, which includes both converging and conflicting results.

There have also been null and inconsistent effects reported of binaural beats on cognition and neurophysiological processes. Ingendoh et al. (2023), for instance, conducted a systematic review and concluded that, despite the occurrence of positive trends, there is still unclear evidence of brain oscillation entrainment with the use of binaural beats, especially in heterogeneous populations as well as in diverse settings^19^. Neither was there a significant modulation of adult attention with gamma-frequency binaural beats reported by Leistiko et al. (2024), which might indicate that the effects of cognition might be age-dependent and state-dependent, or dependent upon the profile of the subject’s neural function^32^.

Also, Baseanu et al. (2024) reported inconsistent findings in trials of mood and anxiety disorders with the risk of placebo response, particularly in psychologically vulnerable populations. Such inconsistencies further justify the need to use standard protocols, controlled conditions, and strict designs in an honest attempt to ascertain the real efficacy of binaural beats^22^.

Despite the positive findings of this research, there are many limitations that need to be highlighted. One of the main limitations is the relatively small size of the sample, which can taint the generalizability of the findings. The research only tested a particular rate of the binaural beat, and the effect of other rates or their interaction was not tested.

Based on the reality that the effects of the binaural beats can become different across varying frequency ranges, more extensive research on the diverse rates and their potential effects is needed. EEG coherence was also used, but not structural or deep-network-level imaging protocols (e.g., fMRI or MEG) to indicate the underlying neural processes affected by the stimulation. The study was also conducted with the testing of measurement of phonological awareness, but not the identification of the neural changes as related to improved real-world reading ability, fluency, and academic success.

Future research should aim to address the limitations mentioned above by incorporating larger and more diverse participant groups to improve the generalizability of the results. Expanding the demographic diversity of the sample, including factors such as age, gender, and cultural background, will help determine whether the effects of binaural beats are consistent across different populations. In terms of methodology, utilizing advanced neuroimaging techniques, such as functional MRI, would allow for a deeper understanding of the neural mechanisms underlying the effects of binaural beats on dyslexic children.

On the other hand, the technology in the field of auditory neuromodulation is advancing rapidly. Portable EEG, customized neurofeedback, and pattern recognition using AI can make the use of binaural beats in the rehabilitation of cognition more specific, adaptive, and scalable. Combining this modality with other treatment strategies, including language rehabilitation, improvement of executive function, and even virtual reality, may significantly improve outcomes.

## Methods

This study aimed to explore the cognitive effects of binaural beats on phonological awareness and cortical connectivity in dyslexic children, without any intent to provide therapeutic intervention or clinical treatment. Binaural beats were employed solely as non-invasive auditory stimuli in this exploratory cognitive research, consistent with their use in psychological and neuroscience studies to investigate perceptual and neural phenomena^33–35^. The research was designed as a controlled, double-blind, randomized exploratory investigation to minimize bias and enhance scientific validity. Participants were randomly assigned to groups receiving either beta or theta binaural beats or a control condition, with neither the participants nor the assessors aware of group allocation. The control condition consisted of isochronous tones without frequency differences, ensuring no perceptual beat illusion or potential physiological effects.

Before the first session, all participants and their legal guardians were fully informed about the non-invasive, non-therapeutic, and purely exploratory nature of binaural beats, which were used exclusively as auditory stimuli rather than any form of clinical intervention. Informed consent was obtained from all participants and their legal guardians in accordance with ethical guidelines. The study protocol was approved by the Shahid Beheshti University Ethics Committee (Reference No: IR.SBU.REC.1403.052), which explicitly confirmed that the study does not constitute a clinical trial as defined by the International Committee of Medical Journal Editors (ICMJE) due to its non-therapeutic, non-invasive, and exploratory design focused on cognitive observation rather than health outcomes. All procedures adhered to the ethical standards of the Helsinki Declaration and relevant institutional regulations.

To assess the potential effects of beta and theta binaural beats on cognitive measures, linguistic and electrophysiological evaluations were conducted by blinded assessors. Pre-test, post-test, and follow-up assessments were performed to measure school performance, intelligence, auditory and visual attention, and phonological awareness. Post-testing occurred after the completion of twelve binaural beats sessions, with follow-up assessments conducted six weeks later. All procedures were non-invasive and designed to ensure participant safety, data validity, and minimization of experimental bias, aligning with standard practices in cognitive neuroscience research.

### Participants

Forty-five dyslexic children between 6.5 and 8.3 years of age (19 girls and 26 boys) were selected. Their full-scale WISC-4 IQ mean and standard deviation were 109 and 6.16, and the Verbal Comprehension Index (VCI) ranged from 89 to 101. All dyslexic children went to regular schools. The diagnosis of dyslexia had been made by a psychiatrist utilizing the Dyslexia Assessment Test (DAT), a test often used in Iran to evaluate children with dyslexia. Children with a reading accuracy and fluency score in the lowest 10% for their age group were found to be dyslexic in terms of this test, and so were differentiated from normally developing readers.

None of the subjects demonstrated mental retardation, cerebral palsy, or other overt neurological signs.

All participants in the study spoke Persian as their first language. All children were right-handed. Handedness was assessed by a classification of hand preference^36^.

For this study, the inclusion criteria of dyslexia are a substandard reading performance combined with at least an average intelligence (IQ ≥ 85) and having normal hearing, too.

Exclusion criteria included a personal or family history of mental illness, brain injury, neurological disorder, serious medical condition, epilepsy, and a family history of genetic disorder.

### The participants were assigned to the following groups

One group consisted of fifteen dyslexic children who took the beta band frequency of the binaural beat (L = 240 Hz, R = 255 Hz). The second group included fifteen dyslexic children who took theta band frequency of binaural beat (L = 240 Hz, R = 245 Hz), and the last group consisted of fifteen dyslexic children who received isochronous tones (R: 240 Hz, L: 240 Hz).

Participants were asked to refrain from any caffeinated food items for at least 4 h before testing and to get an average night’s sleep. Compliance was confirmed by self-reporting and by asking about their duration of sleep.

All subjects voluntarily gave written informed consent and were informed of the test procedure.

Subjects were seated in a sound and light-attenuated room, controlled at an ambient temperature of 22 degrees centigrade.

### Language, attention, and intelligence tests

The group of children with dyslexia was submitted to a language test to investigate the correlation between EEG and neurolinguistic findings of dyslexia. The included test measured tasks related to phonological awareness. This test was assessed at the first and the last sessions of the binaural beat sessions and at follow-up.

The Persian PA(phonological awareness) test was used to assess phonological awareness skills at the pre-test, post-test, and follow-up measurements^37,38^.

For the general intelligence assessment, the WISC-4 was carried out at the first measurement (pre-test) session.

To assess the subject’s auditory and visual attention, the IVA-2 test was taken. The range of auditory attention score was from 85 to 110, and for visual attention was from 83 to 95.

### Binaural beats formation

Multiple acoustic conditions were evaluated during the session. The control condition included isochronous tones (R = 240 Hz, L = 240 Hz). Experimental conditions included (1) a 5 Hz binaural beat as theta (L = 240 Hz, R = 245 Hz), and (2) a 15 Hz binaural beat as beta (L = 240 Hz, R = 255 Hz). The tones presented to the right and left ears are indicated by R and L, respectively. The tones were created in Audacity software, played through stereo headphones (MDR-NC7, Sony), and set by the participants at the start of the session to a comfortable load level while the participant was seated comfortably in a chair.

**Fig. 15:**
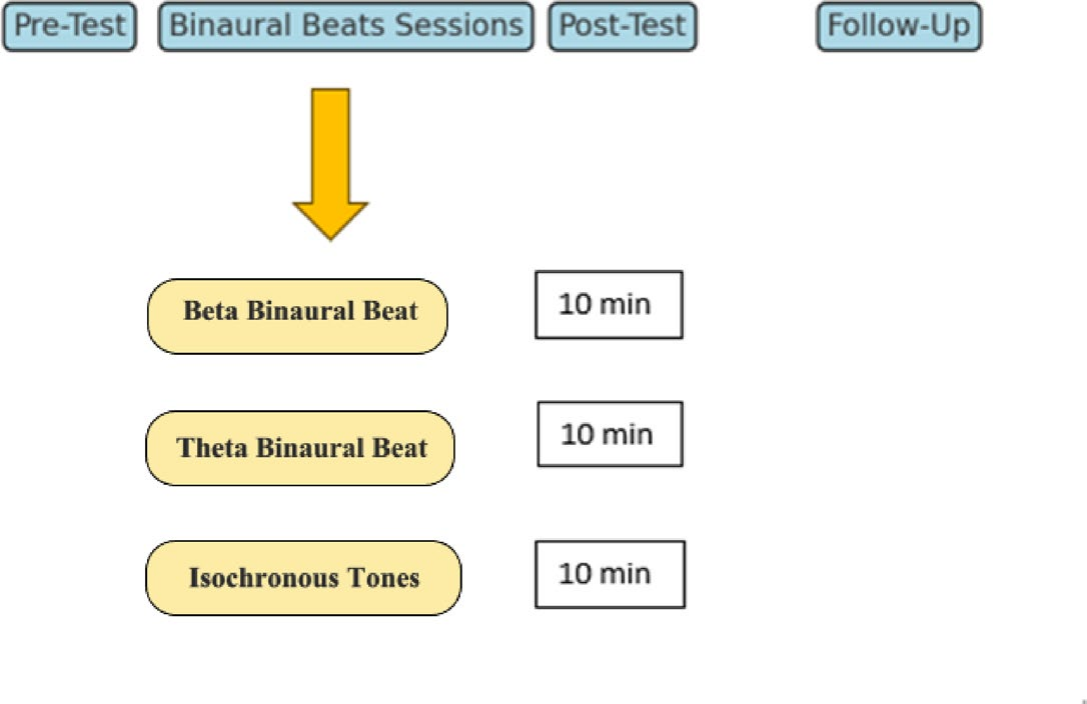
Experimental design overview.

The output sound pressure level (SPL) of the stimulus was normalized to the 73 dB level to match the optimal level of auditory binaural beats, and volume was kept at a constant level throughout the sessions. All participants confirmed that the SPL used would not distract them and described the sound level as comfortable^39^. The binaural beat auditory sessions for all three groups were 12 sessions, 3 times a week, and the duration of each session was 10 min (Fig. 15).

### Electroencephalographic and data acquisition

Participants were seated in a sound and light-attenuated room, controlled at an ambient temperature of 22◦C.

A resting-state EEG was recorded in eyes-closed and eyes-open conditions for three minutes. The duration has been chosen following well-established standards in neurophysiology to have enough data for reliable spectral and connectivity analysis. Previous research has indicated that at least three minutes of unbroken EEG recording is sufficient to obtain representative and stable patterns of neural activity and reduce fluctuations in brain signals. Both conditions facilitate a complete analysis of cortical dynamics because resting-state oscillatory activity changes widely between eyes-closed and eyes-open states, particularly in alpha and theta frequency bands^40^. Subjects were asked to sit quietly and keep their eyes open; participants were asked to fix their eyes on a red dot presented on a computer screen. EEG was recorded from each of the subjects employing the 10–20 montage and ordinary silver chloride skin electrodes. EEG data were acquired from 19 channels (Quik cap; NuAmps;10–20 electrode international system: FP1, FP2, F3, F4, F7, F8, Fz, C3, C4, Cz, P3, P4, Pz, T3, T4, T5, T6, O1, O2), and with two earlobes’ re-reference channels (A1, A2), and with a ground at Cz. Skin resistance was < 10 kΩ for all electrodes. The sampling rate of all channels was 250 Hz, with a 30 Hz low-pass filter and a 0.5 Hz high-pass filter to remove drift and the 60 Hz power line noise. The EEG was recorded in three phases: first, before the start of the binaural beat sessions with binaural beats to establish a baseline of the electrophysiological state of the subjects, after the last binaural beat session with binaural beats, and lastly, in the follow-up period. Six weeks after the final binaural beats session, the follow-up was conducted to ascertain the enduring effects of binaural beats, ascertain any new changes in phonological awareness, and the electrophysiological status of the brain, and compare the results with the initial baseline to ascertain sustained change or return to the pre-state.

### EEG signal preprocessing and Functional connectivity analyses

After the raw EEG signal is obtained, the next step is to do pre-processing. The NeuroGuide can automatically select the good EEG signal by Edit- > Automatic Selection. It detects a similar pattern from the ten-second template, which was chosen before, to determine good parts of the EEG signal. This pre-processing is done by NeuroGuide Inhouse 3.2.1(NeuroState 3.2.1) software.

For processing the data, we have focused on brain connectivity measured by standard intrahemispheric and interhemispheric coherence in Neuroguide software. Processing of the cleaned signal is started by generating a report from the NeuroGuide software containing the desired information of the EEG signal, such as coherence.

### Statistical analyses

Statistical analyses were conducted to evaluate the effects of theta and beta binaural beats on phonological awareness and cortical connectivity measures.

Descriptive statistics (means ± standard deviations) were calculated for all variables at pre-test, post-test, and follow-up.

Normality was assessed separately for EEG coherence measures and phonological awareness scores using the Shapiro–Wilk test, and homogeneity of variances for EEG data was evaluated using Levene’s test. Baseline equivalence among groups at pre-test was examined for both phonological awareness scores and EEG coherence measures using the Kruskal–Wallis test.

EEG coherence data met parametric assumptions and were therefore analyzed using a mixed-design repeated-measures ANOVA, with Time (pre-test, post-test, follow-up) as the within-subjects factor and Group (beta binaural beat, theta binaural beat, control) as the between-subjects factor. Significant interactions were followed by post hoc comparisons with False Discovery Rate (FDR) correction to control for multiple comparisons across frequency bands and electrode pairs. Effect sizes are reported as partial eta squared (η^2^p). Although raw EEG coherence values met parametric assumptions, normality of coherence change scores (post–pre) was assessed separately and was not consistently satisfied; therefore, non-parametric tests were used for between-group comparisons of change scores.

Phonological awareness scores did not meet normality assumptions and were therefore analyzed using non-parametric tests. Within-group changes across time were examined using the Friedman test.

Between-group differences in phonological awareness improvement were assessed using the Kruskal–Wallis test on change scores (post-test minus pre-test), followed by Dunn–Bonferroni post hoc comparisons where appropriate.

Pearson correlation analyses were conducted within each group to examine associations between changes in EEG coherence and phonological awareness scores. Correlation coefficients (r), p-values, and sample sizes are reported, and FDR correction was applied to account for multiple correlations.

All statistical analyses were performed using SPSS version 27, with a significance threshold of p < 0.05.

## Data Availability

The datasets generated during and/or analyzed during the current study are available from the corresponding author on reasonable request.
